# Probing the coordination and function of Fe_4_S_4_ modules in nitrogenase assembly protein NifB

**DOI:** 10.1038/s41467-018-05272-8

**Published:** 2018-07-19

**Authors:** Lee A. Rettberg, Jarett Wilcoxen, Chi Chung Lee, Martin T. Stiebritz, Kazuki Tanifuji, R. David Britt, Yilin Hu

**Affiliations:** 1 Department of Molecular Biology and Biochemistry, University of California, Irvine, CA 92697-3900 USA; 2 Department of Chemistry, University of California, Davis, CA 95616 USA

## Abstract

NifB is an essential radical *S*-adenosylmethionine (SAM) enzyme for nitrogenase cofactor assembly. Previous studies show that NifB couples a putative pair of [Fe_4_S_4_] modules (designated K1 and K2) into an [Fe_8_S_9_C] cofactor precursor concomitant with radical SAM-dependent carbide insertion through the action of its SAM-binding [Fe_4_S_4_] module. However, the coordination and function of the NifB cluster modules remain unknown. Here, we use continuous wave and pulse electron paramagnetic resonance spectroscopy to show that K1- and K2-modules are 3-cysteine-coordinated [Fe_4_S_4_] clusters, with a histidine-derived nitrogen serving as the fourth ligand to K1 that is lost upon K1/K2-coupling. Further, we demonstrate that coexistence of SAM/K2-modules is a prerequisite for methyltransfer to K2 and hydrogen abstraction from the K2-associated methyl by a 5′-deoxyadenosyl radical. These results establish an important framework for mechanistic explorations of NifB while highlighting the utility of a synthetic-cluster-based reconstitution approach employed herein in functional analyses of iron–sulfur (FeS) enzymes.

## Abstract (web summary)

NifB is a key enzyme in the biosynthesis pathway of the nitrogenase FeMo cofactor. Here, the authors investigate the maturation of its iron-sulfur clusters by EPR and biochemical analyses, showing how individual precursor clusters participate in the formation of the final iron-sulfur cluster.

## Introduction

Nitrogenase is a complex metalloenzyme that catalyzes the remarkable chemical transformations of N_2_ to NH_3_, and CO to hydrocarbons, at its cofactor site^1–5^. Designated the M-cluster, the cofactor of the Mo-nitrogenase is a [MoFe_7_S_9_C(*R*-homocitrate)] cluster comprising [MoFe_3_S_3_] and [Fe_4_S_3_] subclusters ligated by three µ_2_-sulfides and one µ_6_-interstitial carbide, with an *R*-homocitrate moiety providing further coordination to its terminal Mo atom^6–8^. The unique reactivity of M-cluster has sparked interest in elucidating its biosynthetic mechanism. Such knowledge is crucial for understanding the structure–function relationship of nitrogenase, which in turn could facilitate future development of biomimetic catalysts for ambient conversion of N_2_ and C_1_ substrates into valuable chemical commodities. Previous studies have led to the proposal of a working model of M-cluster assembly (Supplementary Fig. 1)^9–20^. The early stage of this process involves the coupling and rearrangement of two putative [Fe_4_S_4_] clusters (designated the K-cluster) on an assembly scaffold, NifB, concomitant with the insertion of an interstitial carbide and a 9th sulfur, giving rise to a [Fe_8_S_9_C] core (designated the L-cluster) that is nearly indistinguishable in structure from the M-cluster (Supplementary Fig. 1a, i–iii). Subsequently, the 8Fe L-cluster is matured into an M-cluster on another assembly scaffold, NifEN, upon substitution of a Mo-homocitrate conjugate for one terminal Fe atom of the cluster by NifH, the cofactor maturase (Supplementary Fig. 1a, iv). Finally, the M-cluster is transferred to its target binding site in NifDK, the catalytic component of Mo-nitrogenase (Supplementary Fig. 1a, v).

Of all components along the M-cluster assembly pathway, NifB plays a pivotal role in transforming small, putative 4Fe units (K-cluster) into an 8Fe entity (L-cluster) that has the complete FeS core structure in place, as well as the interstitial carbide. A member of the radical *S*-adenosyl-l-methionine (SAM) enzyme family^21,22^, NifB carries the signature CxxxCxxC radical SAM motif, as well as a number of ligands that could accommodate coordination of the entire complement of Fe atoms of the M-cluster^9,10,23^. Characterization of the NifB proteins from *Azotobacter vinelandii*^11–13^ and *Methanosarcina acetivorans*^14^ has unveiled a radical SAM-dependent mechanism employed by NifB for carbide insertion, which begins with methyltransfer in an S_N_2-type mechanism from SAM to a putative [Fe_4_S_4_] cluster pair (Supplementary Fig. 1b, i). Subsequently, the transferred methyl group undergoes hydrogen abstraction by a SAM-derived 5′-deoxyadenosyl (5′-dA•) radical (Supplementary Fig. 1b, ii). This step is followed by further deprotonation and/or dehydrogenation of the resultant carbon radical, which occurs concomitantly with the coupling and rearrangement of the putative[Fe_4_S_4_] clusters pair into an [Fe_8_S_9_C] core via radical chemistry (Supplementary Fig. 1b, iii). Together, these results suggest the presence and concerted action of three 4Fe modules, namely, the two 4Fe modules (designated the K1- and K2-modules) that give rise to the K-cluster and a third 4Fe module that is ligated by the radical SAM motif (designated the SAM-module), on NifB. However, despite the past and recent progress toward elucidating the NifB-catalyzed reaction^11–14,23–27^, questions remain open as to the 4Fe nature of the building blocks used to generate the 8Fe core, the coordination of the different cluster modules in NifB, and the biosynthetic event that occurs on each of these NifB-associated cluster modules.

Here, we show that NifB variants carrying the individual K1-, K2- and SAM-modules can be reconstituted with synthetic [Fe_4_S_4_] clusters, thereby providing conclusive evidence that NifB consists of three distinct 4Fe modules. Continuous wave (CW)- and pulse EPR analyses reveal that both K1- and K2-modules are coordinated by 3 Cys ligands, with a His residue providing an additional nitrogen ligand to the K1 module that is lost upon coupling of the K1- and K2-modules into an 8Fe core; whereas biochemical experiments further demonstrate that coexistence of the SAM- and K2-modules is a prerequisite for methyltransfer to the K2-derived sulfide atom and the subsequent hydrogen abstraction from the K2-associated methyl group by a 5′-dA• radical. These results not only lay an important foundation for further mechanistic investigations of the NifB-catalyzed reactions, but also point to the utility of the synthetic-cluster-based reconstitution protocol in functional analyses of other FeS systems.

## Results

### Establishing the 4Fe nature of the NifB-associated clusters

**Fig. 1:**
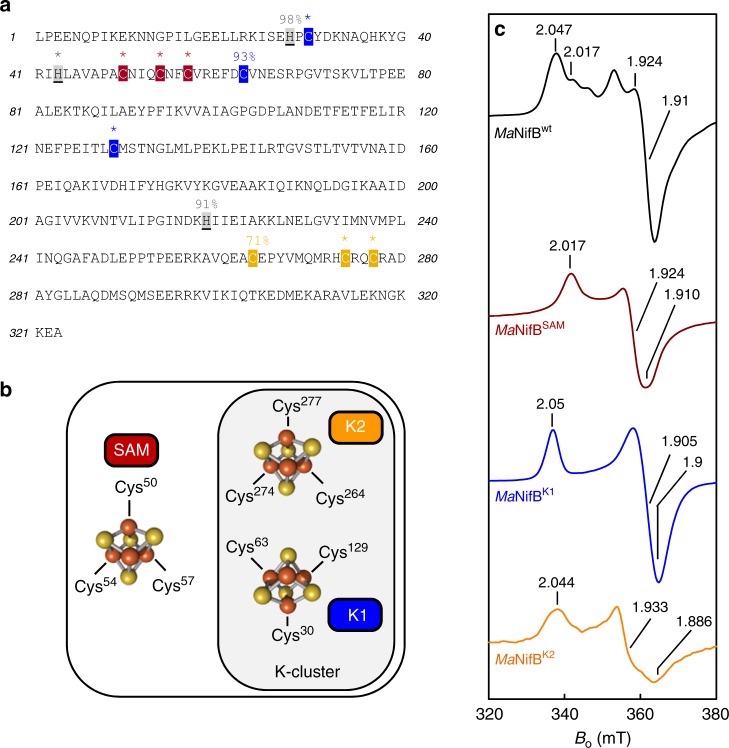
Assignment of three [Fe_4_S_4_] cluster modules in *Ma*NifB. **a** The primary sequence of *Ma*NifB. The Cys and His residues that are conserved among 45 NifB sequences from various organisms^14^ are highlighted, with those 100% conserved indicated with a *, and others noted for the percentages of conservation among these organisms. **b** Schematic presentations of the SAM-, K1-, and K2-modules in *Ma*NifB. A 3-Cys ligation pattern is proposed for all three modules, with the proposed ligands indicated in each module. **c** EPR spectra of the synthetic [Fe_4_S_4_] cluster-reconstituted wild-type *Ma*NifB (*Ma*NifB^wt^, black) and *Ma*NifB variants carrying the SAM (*Ma*NifB^SAM^, brown), K1 (*Ma*NifB^K1^, blue), and K2 (*Ma*NifB^K2^, orange) modules, respectively. The spectra were collected as described in Methods, and the *g* values of each spectrum are indicated. The CW EPR experiment was performed four times. Representative results are shown in **c**. See Supplementary Table 1 for Fe contents, spin concentrations and protein concentrations of EPR samples

A series of variants—each carrying one of the three proposed 4Fe modules—was generated on the template of the NifB protein from *M. acetivorans* (designated *Ma*NifB). Previously, *Ma*NifB was successfully expressed in *Escherichia coli*^14^. Sequence analysis of *Ma*NifB reveals the presence of three groups of highly conserved Cys residues—three Cys residues per group—that could potentially serve as ligands for the SAM-, K1-, and K2-modules of NifB (Fig. 1a, b). Specifically, Cys^50^, Cys^54^, and Cys^57^, which form the canonical CxxxCxxC radical SAM motif, are assigned to the SAM-module; Cys^30^, Cys^63^, and Cys^129^, which are located toward the N terminus of the primary sequence, are assigned to the K1 module; and Cys^264^, Cys^274^, and Cys^277^, which are positioned toward the C terminus of the primary sequence, are assigned to the K2 module (Fig. 1a, b). On the basis of the tentative assignment of the Cys residues, each cluster-binding module of *Ma*NifB can be studied independently by mutating the Cys ligands of the other two modules to Ala. Using this approach, we generated three constructs, each encoding a *Ma*NifB variant with a single SAM (designated *Ma*NifB^SAM^)-, K1 (designated *Ma*NifB^K1^)-, or K2 (designated *Ma*NifB^K2^)-module (see Supplementary Fig. 2, i–iii). We then co-expressed each *Ma*NifB variant with the FeS-assembly machinery, IscSUA, in *E. coli*. Purified *Ma*NifB variants, like their native counterpart, showed a monomeric composition comprising a 38 kDa subunit (Supplementary Fig. 3). However, the cluster contents of the as-purified *Ma*NifB proteins were insufficient for spectroscopic and biochemical analyses. To circumvent this problem, we used a recently developed protocol^28^ to first remove the endogenous FeS clusters of the *Ma*NifB variants and then reconstitute these proteins with a synthetic [Fe_4_S_4_] compound (see Supplementary Fig. 2). Such a reconstitution approach has been successfully applied to identify the source of the 9th sulfur during the cofactor maturation process on *Ma*NifB^28^ without interference of the Fe/S impurities often introduced by the traditional FeCl_3_/Na_2_S-based reconstitution method and, in this particular case, it can be used to conclusively determine the [Fe_4_S_4_] identity of the individual modules in *Ma*NifB. Indeed, upon in vitro reconstitution with this synthetic [Fe_4_S_4_] compound, each of the three *Ma*NifB variants has an *S* = 1/2 electron paramagnetic resonance (EPR) signal that is characteristic of a [Fe_4_S_4_]^+^ cluster when the protein is reduced by dithionite (Fig. 1c; also see Supplementary Table 1 for Fe contents, spin concentrations and protein concentrations of EPR samples). Consistent with their different origins, the simulated spectra have distinct *g* values from one another (*Ma*NifB^SAM^: *g* *=* [2.017 1.924 1.910]; *Ma*NifB^K1^: *g* *=* [2.050 1.905 1.900]; *Ma*NifB^K2^: *g* *=* [2.044 1.933 1.886]; also see Supplementary Fig. 4 and Supplementary Table 2 for simulation parameters), and the contributions of most EPR features of the individual modules can be identified in the spectrum of the wild-type *Ma*NifB (Fig. 1c). These observations are exciting, as they provide strong support for the assignment of the respective Cys residues as the ligands of the three modules in *Ma*NifB while supplying direct proof for the [Fe_4_S_4_] identity of the K1- and K2-modules.

### Probing the ligation patterns of NifB-associated clusters

**Fig. 2:**
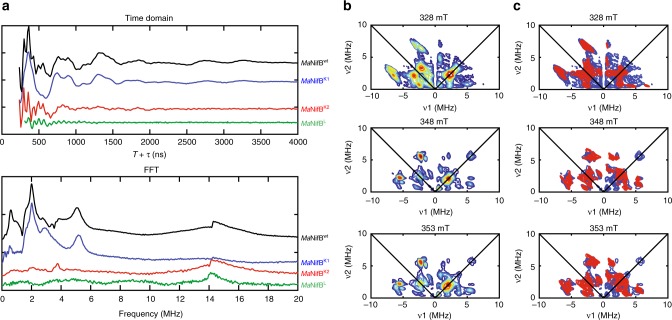
Pulse EPR analysis of *Ma*NifB. **a** Three-pulse ESEEM spectra of dithionite-reduced *Ma*NifB^wt^ (black), *Ma*NifB^K1^ (blue), *Ma*NifB^K2^ (red), and *Ma*NifB^L^ (green). The time domain spectra of *Ma*NifB^wt^ and *Ma*NifB^K1^ have modulations from ^14^N that appear as peaks in the fast Fourier transformed (FFT) spectra between 1 and 8 MHz. The sharp modulations between 250 and 500 ns in the time domain and the resulting broad peak near 14 MHz in the FFT are from nearby weakly coupled protons. **b** HYSCORE spectra of the dithionite-reduced *Ma*NifB^K1^ taken at 328, 348, and 353 mT. **c** The HYSCORE data (blue) can be simulated (red) with a single ^14^N nucleus, with a hyperfine coupling tensor **A** (in MHz) = [2.9 2.9 5.6] and a quadrupole coupling of *e*^2^*Qq*/*h* *=* −2.1 MHz and *η* = 0.4. Three-pulse ESEEM spectra were recorded at 10 K, *τ* = 128–144 ns (values chosen to minimize proton modulations to the spectra), *π*/2 = 12 ns, and a microwave frequency of 9.3366 GHz (*Ma*NifB^wt^, *Ma*NifB^K1^, *Ma*NifB^K2^) or 9.2465 GHz (*Ma*NifB^L^). Spectra were collected near the *g*_2_ value (spectra maxima) for each of the samples (*Ma*NifB^wt^: 343 mT; *Ma*NifB^K1^: 343 mT, and *Ma*NifB^K2^: 344 mT, and *Ma*NifB^L^: 340 mT). *Ma*NifB^K1^ HYSCORE spectra were recorded at 10 K, *τ* = 128–132 ns, and *π*/2 = 12 ns

The ligation patterns of the K1- and K2-modules were further explored by pulse EPR spectroscopy, using the one-dimensional electron spin echo envelope modulation (ESEEM) and two-dimensional hyperfine sub-level correlation (HYSCORE) pulse EPR techniques. In particular, we examined the presence of nitrogen coupling—previously reported for a NifB homolog from a different methanogenic organism^27^—in *Ma*NifB^K1^ and *Ma*NifB^K2^ in order to assign the nitrogen ligand to a specific cluster module. Interestingly, the three-pulse ESEEM data of *Ma*NifB^wt^ and *Ma*NifB^K1^ reveal modulations of a nitrogen coupled to the K1-cluster (Fig. 2a, blue trace). Data collected across the EPR absorption envelope of MaNifB^K1^ (Supplementary Fig. 5), from *g* = 2.050 to *g* = 1.900, show complex and overlapping peaks from a ^14^N nucleus. While these data are rich in information, extracting that information is difficult given the overlap between peaks. In order to further separate out the observed peaks and reliably interpret the ^14^N hyperfine and quadruple couplings, we collected HYSCORE spectra at each of the principal *g* values for the K1 module. The experimental data are simulated exceedingly well with a ^14^N hyperfine coupling tensor (in MHz) of **A** = [2.9 2.9 5.6] and a nuclear quadrupole coupling of *e*^2^*Qq*/*h* *=* −2.1 MHz and *η* = 0.4 (Fig. 2b, c; also see Supplementary Table 3). The overall hyperfine coupling tensor, **A**, can be analyzed as two major components, where **A** = **A**_iso_ + **A**_dip_. The first term (**A**_iso_) is the isotropic Fermi contact term, where **A**_iso_ = (**A**_1_ + **A**_2_ + **A**_3_)/3; and the second term (**A**_dip_) is the through space dipolar coupling tensor, where **A**_dip_ = [−T −T 2T]. We then compared known values of various N ligands to metal centers (Supplementary Table 3) with the HYSCORE simulation parameters. For a nitrogen directly coordinated to a FeS cluster, an isotopic hyperfine of **A**_iso_ = 3–7 MHz and a dipolar coupling of **T** = 0.9–1 MHz are expected, while a nitrogen which is near but not coordinated to the cluster, such as a backbone amide, would have an **A**_iso_ ~1 MHz. Our data suggest the nitrogen we measured (**A**_iso_^K1^ = 3.8 MHz, **T**^K1^ = 0.9 MHz) is in fact directly coordinated to the K1 module^29–39^. Next, we compared the nuclear quadrupole coupling (*e*^2^*Qq*/*h)* values of ^14^N nuclei, which report on the electric field gradient of the ^14^N nucleus and are sensitive to the environment of the nitrogen that is directly bound to an Fe center. While there are numerous EPR measured hyperfine and quadrupole couplings from nitrogenous ligands to Fe sites, a majority of them are from histidine ligated sites, including those found in the various Rieske-type [Fe_2_S_2_] clusters (*e*^2^*Qq*/*h* *=* 1.9–3.5 MHz) where the cluster contains two His ligands, in the mitoNEET [Fe_2_S_2_] clusters (*e*^2^*Qq*/*h* *=* −2.47 MHz) which contain only a single His ligand, and in myoglobin (*e*^2^*Qq*/*h* *=* −2.24 MHz) where the His ligand is axial to the porphyrin-bound Fe center^30–38,40,41^. The only reported hyperfine and quadrupole coupling from a non-histidine ligand is found in the radical SAM enzyme BioB, where Arg^260^ is coordinated to a [Fe_2_S_2_] cluster (*e*^2^Qq/*h* *=* 2.6–2.8 MHz)^29^. The quadrupole coupling we measured (*e*^2^*Qq*/*h* *=* −2.1 MHz) is lower than the reported value for Arg and is in the range of values for a His coordinated cluster; therefore, we assigned the fourth ligand to the K1 module as histidine. Contrary to what we observed in the case of the K1-cluster, we did not detect ^14^N coupling to the K2-cluster (in *Ma*NifB^K2^) and the L-cluster (in *Ma*NifB^L^, or SAM-treated *Ma*NifB^wt^, wherein the K1- and K2-modules are fused into an 8Fe L-cluster upon addition of SAM) by the same EPR techniques (Fig. 2a, red and green traces). However, the observed absence of nitrogen coupling to the K2 module cannot be used to determine if the fourth Fe site of this cluster is open, is occupied by a coordinated water, or is ligated by an amino acid such as Asp or Glu. The absence of nitrogen ligation to the L-cluster, on the other hand, is particularly interesting, as it suggests a conformational rearrangement upon coupling of the K1- and K2-modules into an 8Fe L-cluster.

### Defining the functions of NifB-associated modules

**Fig. 3:**
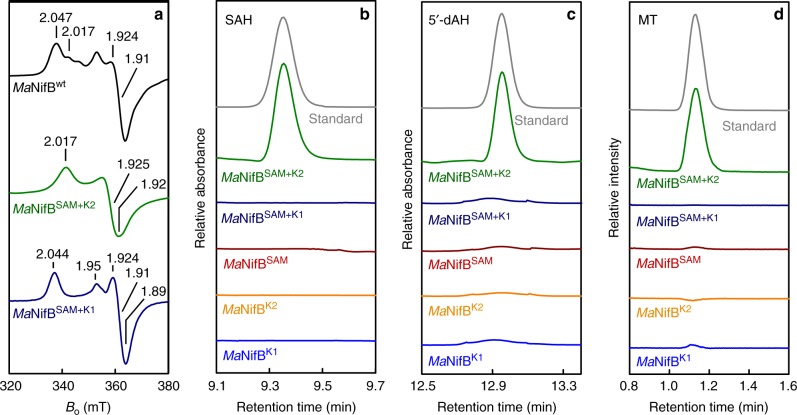
Spectroscopic and functional properties of the cluster modules in *Ma*NifB. **a** EPR spectra of the synthetic [Fe_4_S_4_] cluster-reconstituted wild-type *Ma*NifB (*Ma*NifB^wt^, black) and *Ma*NifB variants carrying SAM plus K2 (*Ma*NifB^SAM+K2^, green) and SAM plus K1 (*Ma*NifB^SAM+K1^, dark blue) modules, respectively. **b**, **c** HPLC elution profiles of SAH (**b**) and 5′-dAH (**c**) standards (gray), and those of SAM incubated with *Ma*NifB variants carrying K1 (*Ma*NifB^K1^, light blue), K2 (*Ma*NifB^K2^, orange), SAM (*Ma*NifB^SAM^, brown), SAM plus K1 (*Ma*NifB^SAM+K1^, dark blue), and SAM plus K2 (*Ma*NifB^SAM+K2^, green) modules, respectively. **d** GC analyses of the methanethiol (MT) standard (gray) and acid-quenched incubation mixtures containing SAM and *Ma*NifB variants carrying K1 (*Ma*NifB^K1^, light blue) and K2 (*Ma*NifB^K2^, orange), SAM (*Ma*NifB^SAM^, brown), SAM plus K1 (*Ma*NifB^SAM+K1^, dark blue), and SAM plus K2 (*Ma*NifB^SAM+K2^, green) modules, respectively. The CW EPR (**a**), HPLC (**b**, **c**), and GC (**d**) experiments were performed three times each. Representative results are shown in the figure. See Supplementary Table 1 for Fe contents and protein concentrations of EPR samples

Having established the presence of three distinct [Fe_4_S_4_] modules in *Ma*NifB, we set out to address the question of which biosynthetic event occurs on each module and, particularly, how the SAM-module—the place where radical SAM chemistry takes place—works with the K1- and K2-modules during the cluster maturation process. Using our modular approach, two additional *Ma*NifB variants (see Supplementary Fig. 2, iv, v), one carrying the SAM- and K1-modules (designated *Ma*NifB^SAM+K1^) and the other carrying the SAM- and K2-modules (designated *Ma*NifB^SAM+K2^), were expressed in *E. coli*, followed by purification and reconstitution with the synthetic [Fe_4_S_4_] compound. Interestingly, the spectrum of neither *Ma*NifB^SAM+K1^ nor *Ma*NifB^SAM+K2^ is a simple add-up of the individual spectra of the respective cluster modules: the *Ma*NifB^SAM+K2^ spectrum resembles the *Ma*NifB^SAM^ spectrum in line-shape, but is somewhat broadened and displayed a new *g*_app_ = 1.92 feature (Fig. 3a, green trace); whereas the *Ma*NifB^SAM+K1^ spectrum resembles the *Ma*NifB^K1^ spectrum in line-shape, but is clearly broadened and displays a distinct *g*_app_ = 1.95 feature in addition (Fig. 3a, blue trace). HPLC analysis further reveals that, like the wild-type *Ma*NifB, *Ma*NifB^SAM+K2^ is capable of cleaving SAM into *S*-adenosyl-l-homocysteine (SAH) and 5′-deoxyadenosine (5′-dAH); in contrast, *Ma*NifB^SAM+K1^, along with *Ma*NifB^SAM^, *Ma*NifB^K1^, and *Ma*NifB^K2^, is unable to generate SAH or 5′-dAH upon incubation with SAM (Fig. 3b, c). This observation suggests that the presence of both SAM- and K2-modules is the prerequisite for both SAM-related reactions to occur during the cluster maturation process, with the K2 module serving as the final location for methyl attachment and the SAM-module supplying a 5′-dA• radical for hydrogen abstraction of the K2-associated methyl group. Consistent with this suggestion, the formation of methanethiol is only detected upon acid quench of an incubation mixture of *Ma*NifB^SAM+K2^ and SAM, pointing to the attachment of SAM-derived methyl group to a sulfide of the K2 module (Fig. 3d).

## Discussion

**Fig. 4:**
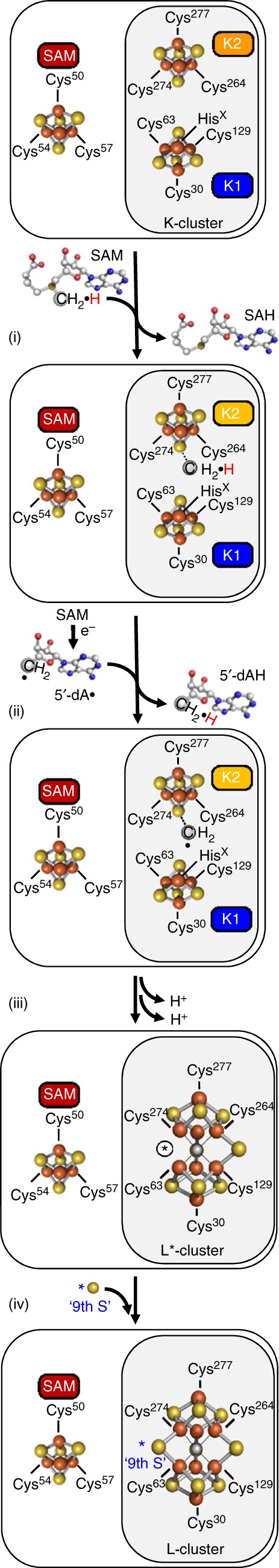
Refined model of L-cluster formation on NifB. This process begins with methyltransfer from one SAM molecule to a sulfide of the K2 module via an S_N_2-type mechanism (i), and it is followed by the formation of a 5′-dA• radical via homolytic cleavage of a second SAM molecule by the SAM-module, and the subsequent hydrogen abstraction from the K2-bound methyl group by 5′-dA• (ii). The resulting, K2-bound methylene (–CH_2_•) radical then initiates the coupling/rearrangement of the K2- and K1-modules into an L*-cluster ([Fe_8_S_8_C]) that resembles the L-cluster ([Fe_8_S_9_C]) except for the absence of a belt sulfur (i.e., the 9th sulfur) (iii). Subsequently, the 9th sulfur is inserted concomitantly with further deprotonation/dehydrogenation of the carbon intermediate until an interstitial C^4−^ ion is generated in the center of a fully assembled L-cluster (iv). These events are accompanied by loss of a conserved His ligand to the K1 module and, possibly, one or more Cys ligands to both the K1- and K2-modules, to accommodate the structural rearrangement of the cluster during this process (iii, iv). This model is proposed based on data from this work and a related work^28^. The encircled black asterisk in the scheme of L*-cluster represents the vacant site in the belt region of the cluster, which may be occupied by a cysteine thiolate or water molecule

A refined pathway of the K- to L-cluster conversion on NifB (Fig. 4) can be proposed based on these results, as well as those from a recent work that traced the origin and insertion of the 9th sulfur of the cofactor^28^. This pathway starts with methyltransfer from one equivalent of SAM to a bridging sulfide of the K2 module via an S_N_2-type mechanism, followed by generation of a 5′-dA• radical via homolytic cleavage of a second equivalent of SAM bound to the SAM-module, and abstraction of a hydrogen atom from the K2-bound methyl group. These events result in a K2-bound methylene (–CH_2_•) radical, which then initiates the coupling/rearrangement of the K2- and K1-modules into an L*-cluster ([Fe_8_S_8_C]) that has the core structure of the L-cluster ([Fe_8_S_9_C]) except for the absence of the 9th sulfur in the belt region of the cluster. Subsequently, the 9th sulfur is inserted concomitantly with further dehydrogenation or deprotonation of the carbon intermediate until a μ_6_-coordinated C^4−^ ion appears in the central cavity of the cluster. Of the various reactions catalyzed by NifB, the S_N_2-type methyltransfer and the subsequent hydrogen abstraction from the methyl are similar to the reactions catalyzed by Cfr and RlmN, two radical SAM enzymes involved in the methylation of rRNA^42–44^.

The cluster transformation process requires structural rearrangement to allow the formation of an L-cluster and the subsequent transfer of the L-cluster from NifB onto the next biosynthetic apparatus, NifEN, along the cofactor assembly pathway (Fig. 4). Central to the structural rearrangement is the loss of the N ligand to the K1 module, as well as possible loss of additional Cys ligands to the K1- and K2-modules. This type of labile N ligand is also observed in mitoNEET, where a single His coordination to a [Fe_2_S_2_] cluster is involved in the release of the cluster to downstream acceptor proteins through a protonation event^45,46^. It is important to note the complexity of the reaction catalyzed by NifB, where there are two substrate molecules of SAM, two substrate [Fe_4_S_4_] clusters (i.e., K1- and K2-clusters), and a catalytic [Fe_4_S_4_] cluster (i.e., the SAM-cluster). These [Fe_4_S_4_] clusters can be sensitive to degradation, especially when there is a non Cys-ligated, open Fe site. Therefore, it is advantageous to have a ligand (like the histidine ligand for the K1-cluster) which, triggered by protonation, can be released from the [Fe_4_S_4_] cluster module to facilitate the formation of the much more stable [Fe_8_S_9_C] L-cluster.

Another interesting point to note is the absence of 5′-dAH formation in the sole presence of the SAM-module, and the absence of SAH formation in the sole presence of the K2 module. While the former could be explained by a lack of K2-bound methyl group that can undergo hydrogen atom abstraction by a 5′-dA• radical, the latter could be accounted for by the absence of a nearby SAM-module that renders the K2-cluster in the correct oxidation state for methyl attachment. A previous study has shown that methyltransfer does not occur when NifB is oxidized or reduced by a weak reductant, suggesting that the K-cluster needs to be poised in a certain redox state to render its associated sulfides more nucleophilic for methyltransfer via an S_N_2-type nucleophilic substitution^13^. It is possible, therefore, that the K2 module needs the presence of at least the SAM-module to be able to accept the methyl group, a scenario highlighting the importance of cross-talk between the cluster modules during the cluster transformation process. Additionally, the fact that the amounts of SAH and 5′-dAH formed in the absence of K1 module are much reduced compared to those in the presence of K1 module further points to a coordination between all three cluster modules to maximize the efficiency of cluster transformation.

While details of the various biosynthetic events on NifB await further investigation, the current study conclusively establishes the presence of three [Fe_4_S_4_] units on NifB and provides useful insights into the coordination and function of each module in the process of K- to L-cluster transformation, taking advantage of the synthetic [Fe_4_S_4_]-cluster-based reconstitution approach that can be applied to the functional analyses of a wide range of other FeS enzymes. The series of variants carrying different combinations of the three modules of NifB could be used to strategically uncouple the different steps in the cluster transformation process, and efforts along this line could be further combined with the identification and perturbation of the His ligand that provides the fourth N ligand to the K1 module in hopes of capturing the intermediary snapshots of this process. Advanced spectroscopic and structural investigations are underway with an ultimate goal to unveil the unique radical SAM chemistry that underscores the complex biosynthetic mechanism of the nitrogenase cofactor.

## Methods

### General information

Unless otherwise specified, all chemicals were purchased from Sigma-Aldrich (St. Louis, MO) and Thermo-Fisher Scientific (Waltham, MA), and all experiments were performed under an Ar atmosphere using Schlenk techniques and a glove box operating at <3 ppm O_2_.

### Cell growth and protein purification

*E. coli* strains expressing His-tagged *Ma*NifB^wt^ (strain YM114EE), *Ma*NifB^SAM^ (strain YM163EE), *Ma*NifB^K1^ (strain YM165EE), *Ma*NifB^K2^ (strain YM166EE), *Ma*NifB^SAM+K1^ (strain YM180EE), and *Ma*NifB^SAM+K2^ (strain YM181EE) were grown in Difco LB medium containing 100 mg L^−1^ ampicillin (BD Biosciences) in a BIOFLO 415 fermenter (New Brunswick Scientific) at a temperature of 37 °C, an agitation of 200 rpm and an airflow of 10 L min^−1^. When cell density (measured at OD_600_) reached 0.5, 25 µM IPTG was added to the cell culture to induce protein expression at 25 °C for 16 h. Subsequently, cells were harvested by centrifugation using a Thermo-Fisher Scientific Legend XTR centrifuge, followed by purification of His-tagged *Ma*NifB proteins using methods adapted from the purification of His-tagged nitrogenase proteins^11^.

### Cluster reconstitution and maturation

The as-isolated wild-type or variant *Ma*NifB protein was treated with 20 mM bathophenanthroline disulfonate, an iron chelator, in a buffer containing 5 mM MgATP, 2 mM dithionite (DT; Na_2_S_2_O_4_), 50 mM Tris–HCl (pH 8) and 500 mM NaCl, followed by incubation at room temperature for 1 h to remove the endogenous FeS clusters associated with the protein. Subsequently, this mixture was diluted with a buffer containing 50 mM Tris–HCl (pH 8) and loaded on a Q Sepharose column (GE Healthcare). The column was then washed with a buffer containing 2 mM DT, 50 mM Tris–HCl (pH 8) and 100 mM NaCl prior to elution of the *Ma*NifB protein with a buffer containing 50 mM Tris–HCl (pH 8). Reconstitution of the wild-type or variant *Ma*NifB protein with synthetic [Fe_4_S_4_] clusters (designated [Fe_4_S_4_]^Syn^)^26^ was carried out by adding a dimethylformamide (DMF) solution of [Fe_4_S_4_]^Syn^ dropwise at a molar ratio of 5:1 to the *Ma*NifB protein in a buffer containing 2 mM DT, 20 mM β-mercaptoethanol, 50 mM Tris–HCl (pH 8), and 500 mM NaCl, with continuous stirring on ice. After incubation on ice for 1 h, the reaction mixture was diluted with a buffer containing 2 mM DT and 50 mM Tris–HCl (pH 8) and loaded on a Q Sepharose column. The column was then washed with a buffer containing 2 mM DT, 50 mM Tris–HCl, and 100 mM NaCl prior to elution of the reconstituted *Ma*NifB with a buffer containing 2 mM DT, 50 mM Tris–HCl (pH 8), and 500 mM NaCl. The *Ma*NifB^SAM^, *Ma*NifB^K1^, *Ma*NifB^K2^, *Ma*NifB^SAM+K1^, and *Ma*NifB^SAM+K2^ variants were used as they were for EPR analysis, whereas *Ma*NifB^wt^ was subjected to SAM treatment using an established protocol^14^, during which process its K-cluster was matured into an L-cluster, prior to EPR analysis of the resultant protein species (designated *Ma*NifB^L^).

### Iron determination

The iron concentrations of the wild-type and variant *Ma*NifB proteins were determined by inductively coupled plasma optical emission spectroscopy (ICP-OES) using a Thermo Scientific iCAP7000. Calibration was made by using standard solutions generated from dilution of a 1 mg mL^−1^ stock solution of elemental iron (Inorganic Ventures). Subsequently, each protein sample was mixed with 100 µL concentrated sulfuric acid (H_2_SO_4_) and 100 µL concentrated nitric acid (HNO_3_), and the mixture was heated at 250 °C for 30 min. This procedure was repeated until the mixture became colorless, followed by cooling of the mixture to room temperature, and dilution of the mixture to a total volume of 10 mL with 2% HNO_3_ prior to sample analysis.

### SAM cleavage assays

Each SAM cleavage reaction contained 25 mM Tris–HCl (pH 8), 5% glycerol (v/v), 40 µM *Ma*NifB, and 0.3 µM SAM in a total volume of 0.3 mL. The reaction mixture was incubated at 25 °C for 60 min with intermittent mixing and terminated by filtration through Amicon Ultra 30,000 MWCO centrifugal filters. Subsequently, trifluoroacetic acid (TFA) was added to the reaction mixture at a concentration of 0.14%, followed by analysis of the resultant sample by a Thermo Scientific Dionex Ultimate 3000 UHPLC system, equipped with an Acclaim 120 C18 column (4.6 × 100 mm, 5-µm particle size). The column was equilibrated with 98% buffer A (50 mM KH_2_PO_4_, pH 6.6) and 2% buffer B (100% methanol) before each sample injection (100 µL per sample) for at least 5 min. Following sample injection, a linear gradient of 2–60% buffer B was applied to the column for 20 min, followed by an isocratic flow with 60% buffer B for 8 min, and a linear gradient of 60–2% buffer B for 4 min. Throughout the run, the flow rate of buffer was kept at 0.5 mL min^−1^, and the column was kept at 30 °C. Elution of products was monitored at a UV wavelength of 254 nm.

### EPR analysis

Samples were prepared in a Vacuum Atmospheres glove box with less than 1 ppm O_2_ and flash frozen in liquid nitrogen before analysis. Reduced samples contained 50 mM Tris–HCl (pH 8), 500 mM NaCl, and 2 mM DT. CW EPR spectra were recorded by an ESP 300 E_z_ spectrophotometer (Bruker) interfaced with an ESR-9002 liquid-helium continuous-flow cryostat (Oxford Instruments) using a microwave power of 5 mW, a gain of 5 × 10^4^, a modulation frequency of 100 kHz, and a modulation amplitude of 5 G. Five scans of perpendicular-mode EPR were recorded at 20 K using a microwave frequency of 9.62 GHz.

For pulse EPR analysis, reaction mixtures were transferred into 4 mm (X-band)- or 2 mm (Q-band)-diameter tubes, flash frozen as above, and stored in liquid nitrogen after freezing. All pulse EPR studies were carried out at the UC Davis CalEPR center, using a Bruker EleXsys E580 pulse EPR spectrometer equipped with an Oxford-CF935 liquid-helium cryostat and an ITC-503 temperature controller. Pulse data were collected using a Bruker MS5 probe (X-band) or an R.A. Isaacson-designed cylindrical TE011 resonator (Q-band)^47^ adapted for pulse EPR in an Oxford Instruments CF935 cryostat. Two-pulse field swept (2PFS) EPR spectra were collected using the sequence *π*/2-*τ*-*π*-*τ*-echo, stepping the field after each point. Three-pulse ESEEM spectra were collected using the pulse sequence *π*/2-*τ*-*π*/2-T-*π*/2-*τ*-echo where the delay time, *T*, was increased by 16 ns steps. ESEEM spectra were recorded at 10 K, *τ* = 128–144 ns (values chosen to minimize proton modulations to the spectra), *π*/2 = 12 ns, and a microwave frequency of 9.3366 GHz (*Ma*NifB^wt^, *Ma*NifB^K1^, *Ma*NifB^K2^) or 9.2465 GHz (*Ma*NifB^L^). Spectra were collected near the *g*_2_ value (spectra maxima) for each of the samples (*Ma*NifB^wt^: 343 mT; *Ma*NifB^K1^: 343 mT, and *Ma*NifB^K2^: 344 mT, and *Ma*NifB^L^: 340 mT). Hyperfine sub-level-correlation (HYSCORE) spectroscopy was performed using the pulse sequence: *π*/2-*τ*-*π*/2-T1-*π*-T2-*π*/2-*τ*-echo^48^. In the HYSCORE experiment, T1 and T2 were incremented by 20 ns steps to produce a two-dimensional spectrum. HYSCORE spectra were recorded at 10 K, *τ* = 128–132 ns, and *π*/2 = 12 ns. Spectral processing and simulations were performed using the EasySpin 4.0 toolbox in Matlab R2017b^49^.

All spin quantification was carried out using the program SpinCount^50^. Variable power EPR spectra were collected for *Ma*NifB^SAM^, *Ma*NifB^K1^ and *Ma*NifB^K2^ at 20 K to determine the extent of saturation. All *Ma*NifB samples were found to have a linear response to the microwave power across all values tested. Samples of Cu(II)EDTA between 0.1 mM and 0.5 mM were used as standards for the *S* = 1/2 signals observed for *Ma*NifB variants, at microwave powers between 0.5 and 1 mW. The slope of each spectrum was baseline corrected using the program tools in SpinCount. The double integral was measured across the *S* = 1/2 signal for each spectrum. The integrations were compared between the Cu(II)EDTA standard and the *Ma*NifB samples to determine the spin concentration.

### Acid quench experiments

A published method was adapted for the *Ma*NifB-dependent production of methanethiol^13,51^. The procedure involved removal of excess DT from *Ma*NifB via gel filtration with Sephadex G-25 fine resin that was equilibrated with a buffer containing 25 mM Tris–HCl (pH 8). Subsequently, 40 nmol of *Ma*NifB was mixed with 400 nmol SAM in a total volume of 100 μL in a sealed 300-μL glass vial, incubated for 30 min at 25 °C, and quenched by 25 μL of 1 M HCl. The acid-quenched sample was then incubated at 60 °C for 15 min to release the volatile methanethiol into the headspace, followed by equilibration of the sample to room temperature for 10 min, and injection of the entire headspace by a gas-tight syringe onto a Restek Rxi-1ms column (30 m, 0.32 mm ID, 4 μm df) for analysis by GC–MS (Thermo-Fisher Scientific Trace 1300 GC connected to a Thermo-Fisher Scientific ISQ QD single quadrupole mass spectrometry). During each GC–MS run, the GC inlet and oven temperatures were kept at 30 °C, and the mass spectrometry transfer line and ion source were kept at 250 °C. Using SIM conditions in electron ionization mode, methanethiol was detected at an *m*/*z* ratio of 47.

### Data availibility

The authors declare that all data supporting the findings of this study are available within the article and the Supplementary Information and from the corresponding authors upon reasonable request.

## Electronic supplementary material

Supplementary Information
