# Supplementary material for: Probing the coordination and function of Fe4S4 modules in nitrogenase assembly protein NifB
Source: Nat Commun. 2018 Jul 19;9:2824. doi: 10.1038/s41467-018-05272-8 (PMC6053413; doi:10.1038/s41467-018-05272-8)
Supplement: Supplementary file 1 — Supplementary Information [file 41467_2018_5272_MOESM1_ESM.pdf]

SUPPLEMENTARY INFORMATION

Probing the Coordination and Function of Fe_4S_4 Modules in Nitrogenase Assembly Protein NifB

Lee A. Rettberg,[1,a] Jarett Wilcoxen,[2,a] Chi Chung Lee,[1] Martin T. Stiebritz,[1] Kazuki Tanifuji,[1] R. David Britt,[2]* and Yilin Hu[1]*

[1]Department of Molecular Biology and Biochemistry, University of California, Irvine, CA 92697-3900; [2]Department of Chemistry, University of California, Davis, CA 95616

[a] These authors contributed equally to this work.

*Corresponding authors. Emails: rdbritt@ucdavis.edu; yilinh@uci.edu

SUPPLEMENTARY FIGURES

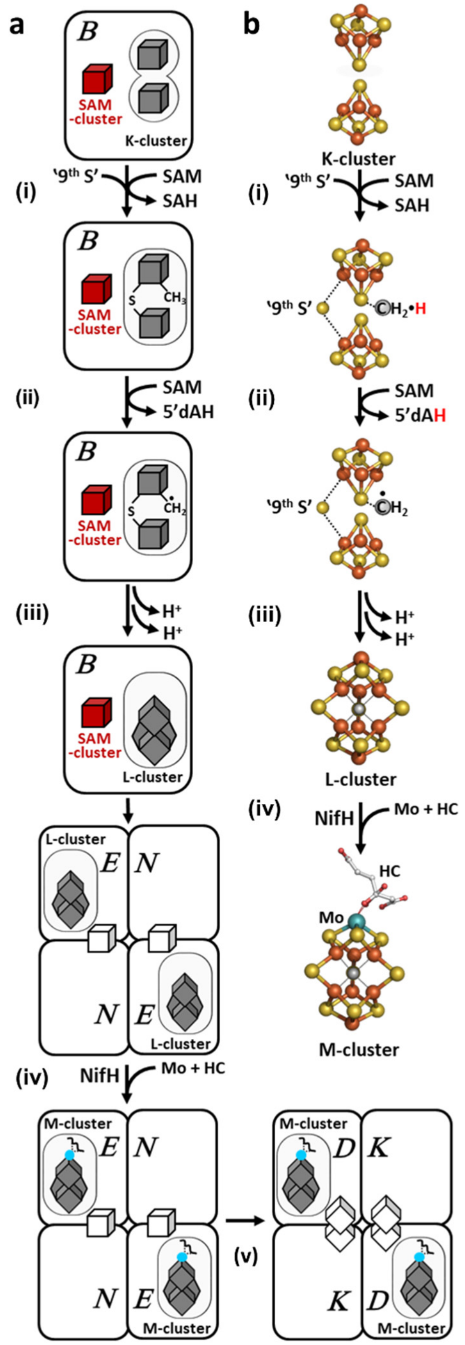

Supplementary Fig. 1 │ Proposed model of M-cluster assembly. (**a**) The assembly of M-cluster involves radical SAM-dependent K- to L-cluster conversion on NifB (i-iii), transfer of L-cluster to NifEN, maturation of L-cluster on NifEN upon insertion of Mo and homocitrate (HC) by NifH (iv), and transfer of the resultant

M-cluster to NifDK (v). (**b**) Coupling of the putative 4Fe units of the K-cluster into an 8Fe L-cluster concomitant with insertion of an interstitial carbide and a 9th sulfur, followed by conversion of the L-cluster to a mature M-cluster ([$MoFe_7S_9C$]) via insertion of Mo and HC. Carbide insertion begins with methyltransfer from SAM to the K-cluster (i) and hydrogen abstraction from the SAM-derived methyl group by 5′-dA• (ii), and continues with further deprotonation/dehydrogenation of the carbon-radical until a carbide appears in the center of the L-cluster (iii). Subsequently, the L-cluster is matured into an M-cluster upon substitution of one terminal Fe atom with Mo and HC (iv). HC, homocitrate

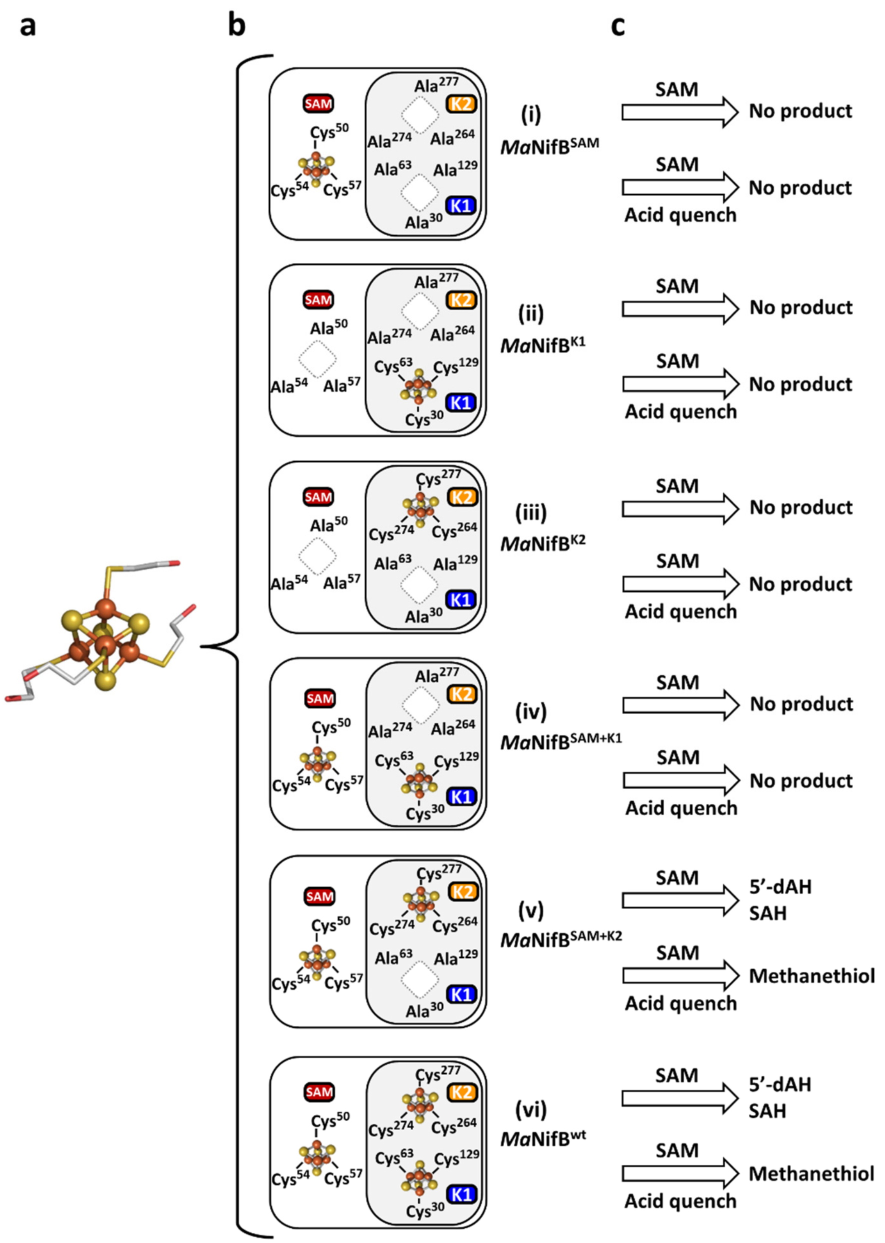

Supplementary Fig. 2 | Schematic presentation of reconstitution of *Ma*NifB proteins by synthetic [Fe_4S_4] clusters. (**a**) Structure of the synthetic [Fe_4S_4] cluster ([$Fe_4S_4(SCH_2CH_2OH)_4]^{2-}$) with exchangeable ligands for protein-bound cysteines[1,2]. Color code: Fe, orange; S, yellow; C, gray; N, blue; O, red. (**b**) Schematic presentation of the *Ma*NifB variants of this study. A 3-Cys ligation pattern is proposed for all three cluster modules (SAM, K1 and K2), with the proposed ligands indicated in each module. The variants carrying individual SAM, K1, and K2 modules (i-iii) or a combination between SAM and K1 or K2 modules (iv, v) were generated by replacing the Cys ligands by Ala. (**c**) Product formation by the wildtype (vi) and variant (i-v) *Ma*NifB proteins upon SAM treatment with or without acid quench.

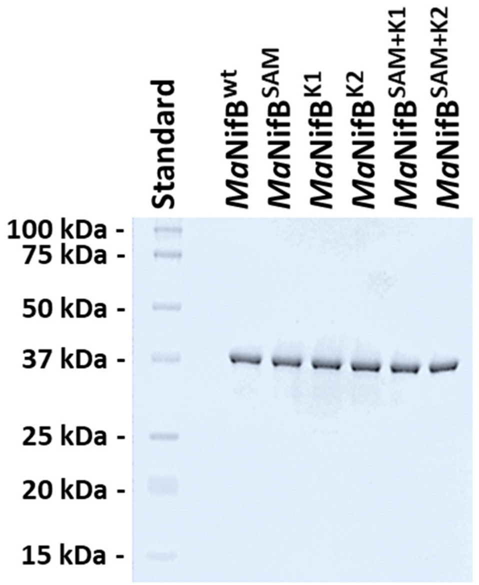

Supplementary Fig. 3 | SDS PAGE of the wildtype and variant *Ma*NifB proteins re-purified after reconstitution by synthetic [Fe_4S_4] clusters[3]. The SDS–PAGE experiment was performed three times. Representative results are shown in the figure.

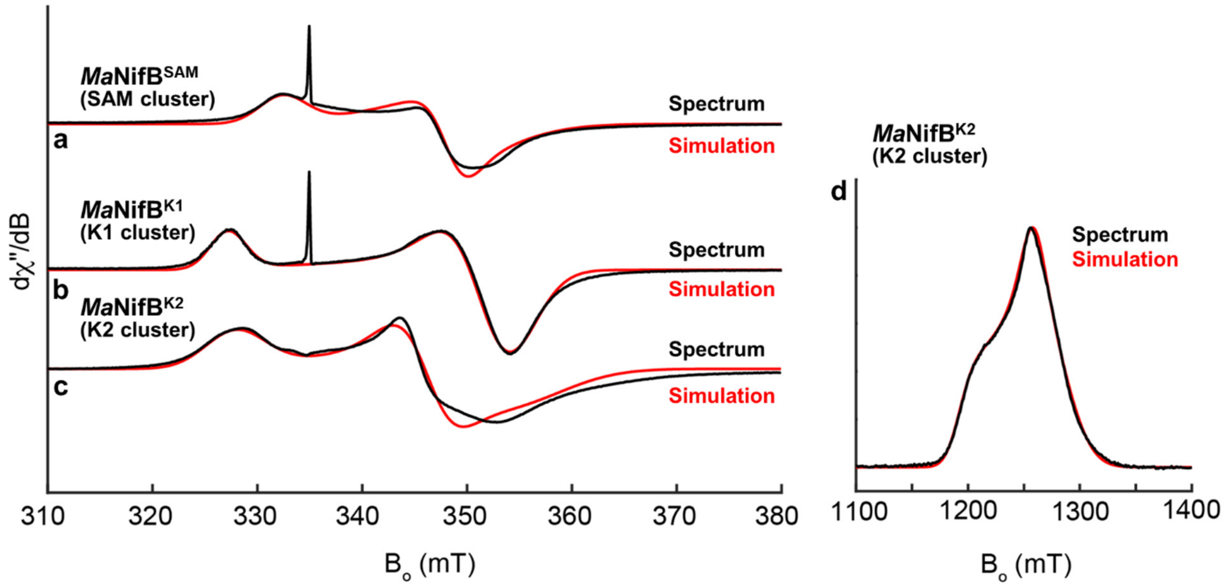

Supplementary Fig. 4│Simulation of the CW and Q-band EPR spectra for single cluster variants of *Ma*NifB using the parameters in Supplementary Table 2. X-band CW EPR spectra of dithionite-reduced (**a**) $Ma\mathrm{NifB}^{SAM}$, (**b**) $Ma\mathrm{NifB}^{K1}$, and (**c**) $Ma\mathrm{NifB}^{K2}$ (black) with simulations of the data (red) using the values in Supplementary Table 2. Data were collected at 10 K, 9.388 GHz microwave frequency, 10 µW microwave power, and 5 G modulation amplitude. (**d**) Q-Band (34.1 GHz) two-pulse field sweep of $Ma\mathrm{NifB}^{K2}$ (black) with simulation of the data (red). Spectra c and d were simulated using the same *g*-values and *g*-strain, with the fit optimized to the Q-band spectrum where a better resolution of *g*-values and pulses allowed for greater selectivity in the presence of contaminating minor species in the background. All spectra above were measured at the CalEPR center at the University of California, Davis. The CW EPR spectra were collected on a Bruker Instruments EleXsys-II E500 CW EPR spectrometer (Bruker Corporation, Billerica, MA) equipped with an Oxford Instruments ESR900 liquid helium cryostat and an Oxford Instruments ITC503 temperature and a gas-flow controller. Samples were measured under non-saturating slow-passage conditions using a Super-High Q resonator (ER 4122SHQE). The Q-band pulse EPR field sweep was collected on a Bruker EleXsys E580 pulse EPR spectrometer equipped with an Oxford-CF935 liquid helium cryostat and an ITC-503 temperature controller, using an R.A. Isaacson-designed cylindrical TE011 resonator adapted for pulse EPR in an Oxford Instruments CF935 cryostat. Two-pulse field swept (2PFS) EPR was collected using the sequence π/2-τ-π-τ-echo, stepping the field after each point. The field swept spectrum was recorded at 10 K, τ = 400 ns, π/2 = 12 ns, and a microwave frequency of 34.10 GHz.

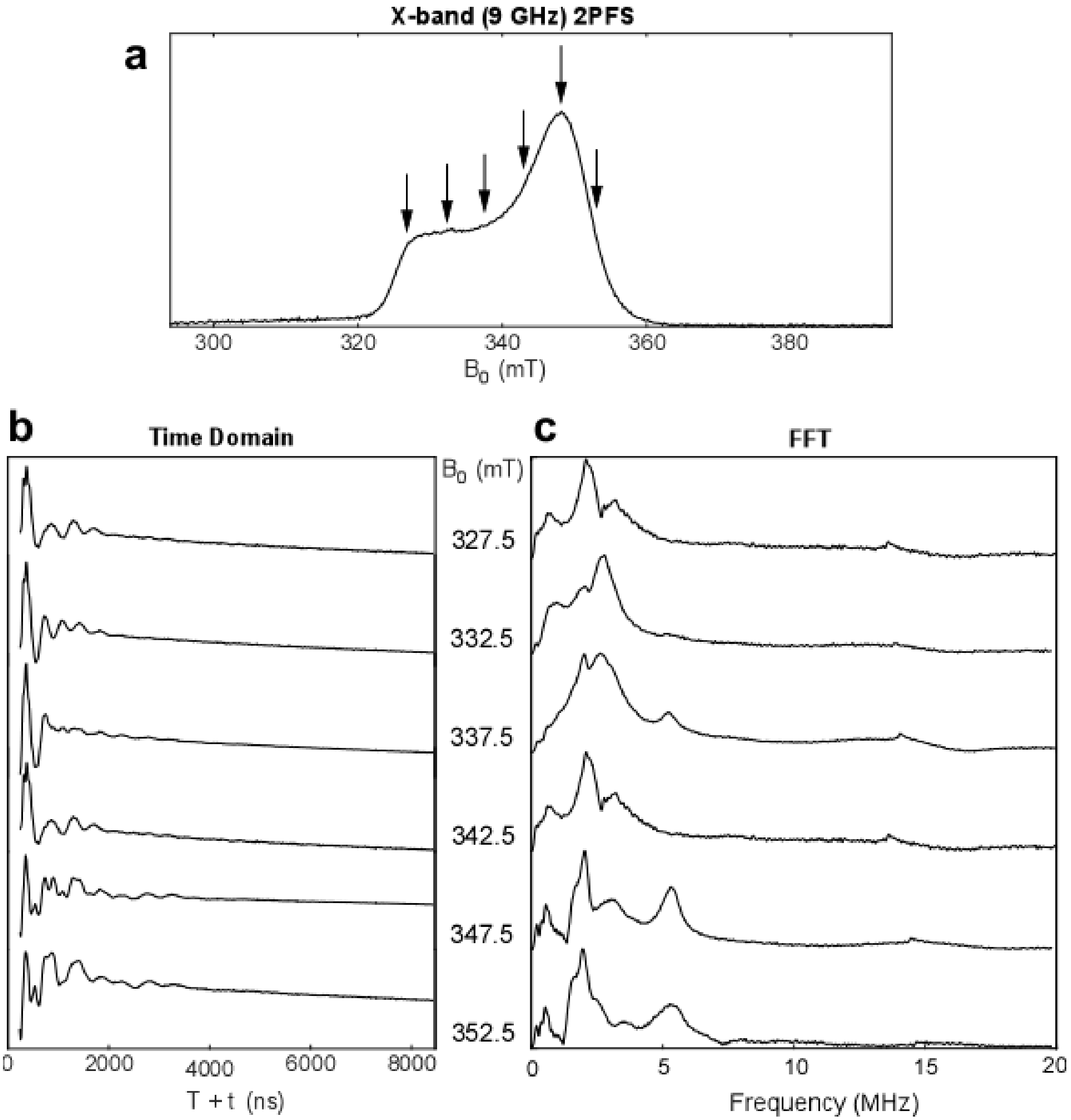

Supplementary Fig. 5 | Field-dependent three pulse ESEEM of *Ma*NifB^{K1}. (**a**) Three pulse ESEEM spectra of dithionite-reduced *Ma*NifB^{K1}, which were recorded at 10 K, τ = 128-144 ns (values chosen to minimize proton modulations to the spectra), $\pi/2$ = 12 ns, and a microwave frequency of 9.3366 GHz. Data were collected across the EPR absorption envelope at the points indicated in the two-pulse field sweep (2PFS) spectrum. (**b**) The time domain spectra of dithionite-reduced *Ma*NifB^{K1}, which have modulations from ^{14}N. (**c**) The fast Fourier transformed (FFT) spectra of of dithionite-reduced *Ma*NifB^{K1}. Modulations from ^{14}N in the time domain spectra (b) appear as peaks in the FFT spectra (c) between 1 and 8 MHz.

SUPPLEMENTARY TABLES

Supplementary Table 1│ Iron content and spin integration

Protein	Fe Content (mol Fe mol^{-1} protein)	(% Occupancy)	Spin Integration (S=1/2 per cluster)	EPR Sample Concentration (μM)
***Ma*NifB^{wt}**	11.9 ± 1.1	99	*nd*	324 ± 29
***Ma*NifB^{SAM+K1}**	6.5 ± 0.7	81	*nd*	300 ± 34
***Ma*NifB^{SAM+K2}**	6.3 ± 0.6	78	*nd*	397 ± 18
***Ma*NifB^{SAM}**	3.1 ± 0.4	78	0.38 ± 0.05	361 ± 16
***Ma*NifB^{K1}**	3.9 ± 0.1	98	1.00 ± 0.03	266 ± 29
***Ma*NifB^{K2}**	2.6 ± 0.4	64	0.30 ± 0.05	255 ± 24

nd = not determined

Supplementary Table 2│Simulation parameters for CW X-band EPR spectra

	g_1	g_2	g_3	σ_1	σ_2	σ_3
**SAM cluster**	2.017	1.924	1.910	0.030	0.020	0.070
**K1 cluster**	2.050	1.905	1.900	0.025	0.035	0.040
**K2 cluster**	2.044	1.933	1.886	0.041	0.029	0.064

Supplementary Table 3│ Hyperfine and quadrupole coupling constants of ^{14}N nitrogen ligated metal centers[a]

	$\mathbf{A}_{iso}$ (in MHz)	**T** (in MHz)	e^2Qq/h (in MHz)	η	Ref[this work,4-18]
*Ma*NifB[b]	3.8	0.9	-2.1	0.4	This Work
Primary aliphatic amine	~1		~4		4
Backbone amide	~1		3-3.4		5,6
BioB Arg^{260}	3.5	0.9	2.6-2.8	0.36-0.54	7
MitoNEET H-87	-6.25	-0.94	-2.47	0.38	8
Riske N1	4.6-5.5		1.9-3.15		9-16
Phtalate dioxygenase N1	6.03	~1	-2.6	0.31	9
Phtalate dioxygenase N2	7.73	~1	-2.3	0.4	9
MoaA-GTP	4		-2.8		17
MoaA-ITP	3		-3.2		17
Myoglobin	13.05	1.6	-2.24	0.45	18

[a] In references where values are reported as a **P** tensor, we have calculated the corresponding e^2Qq/h and η using the following relationships:

$$\mathbf{P} = \frac{e^2Qq}{4I(2I-1)}\begin{bmatrix} -1+\eta \\ -1-\eta \\ 2 \end{bmatrix}$$, where I is the nuclear spin quantum number (I=1 for ^{14}N)

[b] The following Euler angles (in degrees) were used to simulate the HYSCORE spectra (Hyperfine Tensor: α= 0, β = 55, γ = 0; Quadrupole Tensor: α= 0, β = 50, γ = 0)

SUPPLEMENTARY REFERENCES

1. Averill, B. A., Herskovitz, T., Holm, R. H. & Ibers, J. A. Synthetic analogs of the active sites of iron-sulfur proteins. II. Synthesis and structure of the tetra(mercapto-β_3-sulfido-iron) clusters, $(Fe_4S_4(SR)_4)^{2-}$. *J. Am. Chem. Soc.* **95**, 3523−3534 (1973).

2. Barclay, J. E., Davies, S. C., Evans, D. J. & Hughes, D. L. Lattice effects in the Mössbauer spectra of salts of $[Fe_4S_4\{S(CH_2)_nOH\}_4]^{2-}$. Crystal structures of $[PPh_4]_2[Fe_4S_4\{S(CH_2)_nOH\}_4]$ (n=2, 3 and 4). *Inorg. Chim. Acta*. **291**, 101−108 (1999).

3. Tanifuji, K. *et al.* Tracing the '9th sulfur' of the nitrogenase cofactor via a semi-synthetic approach. *Nat. Chem.* **10**, 568–572 (2018).

4. Dikanov, S. A. & Tsvetkov, Y. D. Electron Spin Echo Envelope Modulation (ESEEM) spectroscopy, CRC Press, Inc., Boca Raton, FL. (1992).

5. McCracken, J., Vassiliev, I. R., Yang, E. C., Range, K. & Barry, B. A. ESEEM studies of peptide nitrogen hyperfine coupling in tyrosyl radicals and model peptides. *J. Phys. Chem. B* **111**, 6586−6592 (2007).

6. Yap, L. L., Samoilova, R. I., Gennis, R. B. & Dikanov, S. A. Characterization of the exchangeable protons in the immediate vicinity of the semiquinone radical at the QH site of the cytochrome bo3 from Escherichia coli. *J. Biol. Chem.* **281**, 16879−16887 (2006).

7. Taylor, A. M., Stoll, S., Britt, R. D. & Jarrett, J. T. Reduction of the [2Fe–2S] cluster accompanies formation of the intermediate 9-mercaptodethiobiotin in *Escherichia coli* biotin synthase. *Biochemistry* **50**, 7953−7963 (2011).

8. Dicus, M. M. *et al.* Binding of histidine in the (Cys)3(His)1-coordinated [2Fe−2S] cluster of human mitoNEET. *J. Am. Chem. Soc.* **132**, 2037−2049 (2010).

9. Gurbiel, R. J. *et al.* Electron-nuclear double resonance spectroscopy of ^{15}N-enriched phthalate dioxygenase from *Pseudomonas cepacia* proves that two histidines are coordinated to the [2Fe-2S] Rieske-type clusters. *Biochemistry* **28**, 4861–4871 (1989).

10. Britt, R. D. *et al.* Electron spin echo envelope modulation spectroscopy supports the suggested coordination of two histidine ligands to the Rieske Fe-S centers of the cytochrome b6f complex of spinach and the cytochrome bc1 complexes of *Rhodospirillum rubrum*, *Rhodobacter sphaeroides* R-26, and bovine heart mitochondria. *Biochemistry* **30**, 1892–1901 (1991).

11. Shergill, J. K. & Cammack, R. ESEEM and ENDOR studies of the Rieske iron-sulphur protein in bovine heart mitochondrial membranes. *Biochim. Biophys. Acta* **1185**, 35–42 (1994).

12. Riedel, A.; Fetzner, S.; Rampp, M.; Lingens, F.; Liebl, U.; Zimmermann, J.-L.; Nitschke, W. *J. Biol. Chem*., **270**, 30869–73 (1995).

13. Shergill, J. K., Joannou, C. L., Mason, J. R. & Cammack, R. Coordination of the Rieske-type [2Fe-2S] cluster of the terminal iron-sulfur protein of *Pseudomonas putida* benzene 1,2-dioxygenase, studied by one- and two-dimensional electron spin-echo envelope modulation spectroscopy. *Biochemistry* **34**, 16533–16542 (1995).

14. Dikanov, S. A., Xun, L., Karpiel, A. B., Tyryshkin, A. M. & Bowman, M. K. CW and pulsed EPR characterization of the reduction of the Rieske-type iron-sulfur cluster in 2,4,5-trichlorophenoxyacetate monooxygenase. *J. Am. Chem. Soc*., **118**, 8408–8416 (1996).

15. Gurbiel, R. J. *et al.* Active site structure of Rieske-type proteins: electron nuclear double resonance studies of isotopically labeled phthalate dioxygenase from *Pseudomonas cepacia* and Rieske protein from *Rhodobacter capsulatus* and molecular modeling studies of a Rieske center. *Biochemistry*, **35**, 7834–7845 (1996).

16. Dikanov, S. A., Shubin, A. A., Kounosu, A., Iwasaki, T. & Samoilova, R. I. A comparative, two-dimensional ^{14}N ESEEM characterization of reduced [2Fe-2S] clusters in hyperthermophilic archaeal high- and low-potential Rieske-type proteins. *J. Biol. Inorg. Chem*., **9**, 753–767 (2004).

17. Lees, N. S. *et al.* ENDOR Spectroscopy shows that guanine N1 binds to [4Fe−4S] cluster II of the *S*-adenosylmethionine-dependent enzyme MoaA: mechanistic implications. *J. Am. Chem. Soc.* **131**, 9184–9185 (2009).

18. Scholes, C. P. *et al.* Electron nuclear double resonance (ENDOR) from heme and histidine nitrogens in single crystals of aquometmyoglobin. *J. Am. Chem. Soc.,* **104**, 2724–2735 (1982).
